# Sexual dysfunction associated with selective serotonin reuptake inhibitors in adults with depression: a systematic review and meta-analysis

**DOI:** 10.1007/s00228-026-04011-z

**Published:** 2026-02-21

**Authors:** Sarah Dagostin Ferraz, Leonardo Kuyunga, Peterson Rech, Maria Laura Rodrigues Uggioni, Ana Claudia Rodrigues Candido, Valdemira Santina Dagostin, Fabio Rosa Silva, Tamy Colonetti, Antonio José Grande, Maria Inês da Rosa

**Affiliations:** 1 Translational Biomedicine Laboratory, Graduate Program in Health Sciences, Universidade do Extremo Sul Catarinense (UNESC), Criciúma, SC 88806000 Brazil; 2 Laboratory of Evidence-based Practice, Universidade Estadual de Mato Grosso do Sul (UEMS), Campo Grande, MS Brazil; 3 Instituto Superior Politécnico da CAÁLA, Huambo, Angola

**Keywords:** Depression, Selective serotonin reuptake inhibitors, Sexual dysfunction

## Abstract

**Objective:**

To systematically evaluate the impact of SSRIs on sexual function in adults with depression compared to placebo through a systematic review and meta-analysis of randomized controlled trials (RCTs).

**Methods:**

A systematic search using the terms *“sexual dysfunction”*,* “depression” and “antidepressant”* was conducted in PubMed/MEDLINE, LILACS, Embase, and the Cochrane Library for RCTs published up to June 2025. No language restrictions were applied. Both dichotomous and continuous outcomes were analyzed with 95% confidence intervals using RevMan 5.4. The risk of bias in individual studies was assessed independently by two reviewers using the revised Cochrane Risk of Bias tool for randomized trials (RoB 2).

**Results:**

Thirteen RCTs met inclusion criteria for qualitative synthesis and six were included in meta-analyses. SSRIs were significantly associated with increased risk of orgasmic dysfunction (RR = 3.28, (95% CI of 2.33 to 4.60, *p* < 0.00001; I² = 8%,) and reduced sexual satisfaction (RR = 1.21, (95% CI of 1.11 to 1.32, *p* = 0.0001, I² = 0%,). A non-significant trend toward decreased sexual desire was observed (RR = 1.40, 95% CI: 0.92–2.12, *p* = 0.12; I² = 54%,). No significant differences were detected in total CSFQ scores compared with placebo.

**Conclusion:**

SSRI use is consistently associated with sexual dysfunction, particularly orgasmic dysfunction and reduced sexual satisfaction. The GRADE assessment indicated high to moderate certainty of evidence. Orgasmic dysfunction showed high certainty, with concerns limited to risk of bias. Sexual satisfaction, sexual desire disorders, and CSFQ total scores demonstrated moderate certainty, mainly due to risk-of-bias issues.

## Introduction

Depression is one of the most prevalent and debilitating mental health conditions worldwide. This complex disorder is characterized by persistent mood disturbances, a marked loss of interest in daily activities, chronic fatigue, disrupted sleep and appetite patterns, and significant impairments in cognitive processes and social interactions [1]. The magnitude of this public health challenge is highlighted by data from the World Health Organization, which indicate that approximately 280 million people worldwide were affected by depression in 2021, with prevalence rates of 5% among adults and 5.7% among older adults [2].

The cornerstone of contemporary depression management is largely based on pharmacological interventions, with Selective Serotonin Reuptake Inhibitors (SSRIs) recognized as the gold standard and the most frequently prescribed class of antidepressants in clinical practice [3, 4]. While SSRIs have demonstrated substantial clinical efficacy in alleviating depressive symptoms, their therapeutic benefits are often offset by a range of adverse effects that can significantly compromise treatment adherence and patient quality of life. Among these, sexual dysfunction is one of the most prevalent, distressing, and clinically challenging side effects, frequently serving as a primary reason for treatment discontinuation [5, 6].

The broader spectrum of SSRI-related adverse effects includes gastrointestinal disturbances, such as nausea, diarrhea, or constipation; neurological symptoms, including headaches, insomnia, drowsiness, and tremors; as well as systemic effects, such as dry mouth, excessive sweating, weight fluctuations and sexual dysfunction [7, 8]. A particularly challenging aspect of SSRI therapy is the temporal mismatch between the onset of adverse effects and the manifestation of therapeutic benefits: while side effects typically appear within one to two weeks of treatment initiation, full therapeutic effects generally require two to four weeks to develop, creating a critical period during which patients may experience significant discomfort without perceiving clinical improvement [9].

Sexual function is a key aspect of human well-being, influencing quality of life and psychological health. SSRI-induced sexual dysfunction presents a complex clinical challenge, arising both from antidepressant pharmacology and the underlying depressive disorder. Symptoms include decreased libido, impaired arousal, anorgasmia, delayed ejaculation, erectile dysfunction, and reduced vaginal lubrication, with evidence suggesting a greater impact on women [6, 10]. These dysfunctions are primarily linked to excessive serotonergic activity inhibiting dopaminergic neurotransmission in brain regions essential for motivation, reward, and sexual response regulation [11]. Beyond physiological impairment, sexual side effects are a major contributor to treatment non-adherence, highlighting the critical interplay between sexual satisfaction, mental health, and overall well-being [10].

Despite extensive clinical recognition of the association between SSRI therapy and sexual dysfunction, significant knowledge gaps remain regarding the comprehensive and systematic evaluation of these effects and their clinical implications. In this context, the present study aims to conduct a systematic review of the international literature on sexual function in adults with depression undergoing SSRI treatment, providing a comprehensive synthesis of current knowledge and identifying areas that warrant further investigation.

## Methods

This systematic review was conducted in accordance with the Preferred Reporting Items for Systematic Reviews and Meta-Analyses (PRISMA) guidelines and was prospectively registered with PROSPERO (International Prospective Register of Systematic Reviews) under protocol number CRD42023448691 [12].

The systematic approach employed in this review was designed to comprehensively identify, evaluate, and synthesize the available evidence regarding sexual function in adults with depression receiving SSRI therapy.

### PICOS

The research question was formulated through the PICOS strategy:

- Population (P): Adults aged 18 years or older with depression;
- Intervention (I): Selective serotonin reuptake inhibitors (SSRIs);
- Comparison (C): Placebo or no SSRI usage;
- Outcomes (O): Sexual function (Sexual Symptoms; Sexual Side Effects; Orgasm Dysfunction; Sexual Satisfaction; Sexual Desire);
- Study Type (S): Randomized controlled trials (RCTs).

### Eligibility criteria

The inclusion criteria for this systematic review were designed to capture high-quality evidence while remaining specific to the research question. Studies were eligible if they were randomized controlled trials involving adult participants (≥ 18 years) with a confirmed diagnosis of depression who were not using antidepressant medication at baseline, and in whom selective serotonin reuptake inhibitors (SSRIs) were initiated after randomization in the intervention group. Eligible studies were required to include a comparator group receiving placebo or no SSRI treatment and to explicitly evaluate sexual dysfunction as an outcome. Studies were excluded if they primarily compared sexual dysfunction outcomes between different pharmacological agents rather than evaluating SSRI effects specifically. Additionally, studies involving pregnant or lactating women were excluded to avoid confounding effects from hormonal and physiological changes that could independently influence sexual function outcomes.

### Search strategy

A comprehensive and systematic search strategy was developed to ensure optimal retrieval of relevant literature. The search framework was constructed using three primary keyword domains: “depressive disorder”, “Serotonin Uptake Inhibitors”, “Sexual Dysfunctions”, and “Randomized Controlled Trial” each supplemented with their corresponding synonyms and related terms systematically retrieved from the Medical Subject Headings (MeSH) database to enhance search comprehensiveness and capture variations in terminology across different studies and time periods.

The Boolean search logic employed strategic combinations of these terms using “OR” operators within each conceptual domain to maximize sensitivity, followed by “AND” operators between domains to ensure specificity and relevance to the research question. This approach facilitated the identification of studies that addressed the intersection of all three key concepts while accommodating variations in indexing and terminology preferences across different publications.

The literature search was conducted across multiple comprehensive databases to ensure broad coverage of the available evidence base: Medline (PubMed), LILACS, Embase, and the Cochrane Library for systematic reviews and controlled trials. The temporal scope encompassed all studies published until June 2025, providing a comprehensive historical perspective on the evolution of knowledge in this field.

To optimize the relevance and applicability of findings, the search was appropriately restricted to studies conducted on human subjects, thereby excluding animal studies and in vitro research that would not directly inform clinical practice. Importantly, no language restrictions were imposed during the search process, reflecting a commitment to capturing global research contributions and minimizing potential publication bias associated with language preferences in academic publishing.

### Study selection

The study selection process was implemented through a rigorous, multi-stage screening approach designed to ensure objectivity, minimize selection bias, and maintain methodological transparency. Two independent reviewers (L.K. and T.C.) conducted a comprehensive evaluation of titles and abstracts for all articles identified through the systematic database searches. This dual-reviewer approach served as a critical quality control measure, ensuring that potential studies were evaluated from multiple perspectives and reducing the likelihood of inadvertent exclusion of relevant research.

The screening process was facilitated through the Rayyan web-based platform (http://rayyan.qcri.org), a specialized systematic review management tool that enables efficient collaboration between reviewers while maintaining blinding during the initial screening phases. This digital platform enhanced the standardization and documentation of the selection process, providing a transparent audit trail of all screening decisions.

Following the initial title and abstract screening phase, studies that did not meet the predetermined eligibility criteria were systematically excluded from further consideration. The remaining potentially eligible studies underwent a comprehensive full-text evaluation, again conducted independently by the same two reviewers (L.K. and T.C.), who assessed each study’s complete methodology, results, and conclusions against the established inclusion criteria for this systematic review.

To ensure the integrity and reliability of the selection process, any discrepancies or disagreements between the two primary reviewers regarding study inclusion were systematically resolved through consultation with a third independent review author (M.I.R.), who served as an arbitrator in cases where consensus could not be achieved through discussion. This hierarchical approach to conflict resolution ensured that all selection decisions were made through rigorous peer review and maintained the systematic review’s methodological standards.

### Data extraction

Following the completion of the full-text review and final study selection, a systematic data extraction process was implemented to ensure comprehensive and standardized collection of relevant information from all included studies. Two researchers (L.K. and T.C.) independently performed data extraction from each included study, maintaining the dual-reviewer approach established during the selection phase to minimize extraction errors, reduce potential bias, and enhance the reliability of the collected data.

The data extraction process was structured through the utilization of a standardized collection form specifically designed to capture all essential elements required for comprehensive analysis and synthesis. This systematic approach ensured consistency across all included studies and facilitated subsequent data analysis and interpretation. The standardized extraction form encompassed the following key domains:

#### Study characteristics and methodology

Author identification and publication year, clearly stated research objectives and hypotheses, detailed participant demographics and clinical characteristics, and comprehensive methodological parameters including SSRI dosage regimens, treatment duration, and specific assessment scales or instruments utilized for measuring sexual function and depressive symptoms.

#### Outcomes and findings

Primary and secondary outcome measures as defined by the original authors, detailed presentation of quantitative and qualitative results, and author conclusions and clinical implications as stated in the original publications.

This systematic data extraction approach ensured that all relevant information necessary for comprehensive analysis, synthesis, and interpretation of findings was captured uniformly across all included studies, thereby facilitating robust evidence synthesis and minimizing the risk of important data omission or misinterpretation.

### Risk of bias assessment

The risk of bias in individual studies was assessed independently by two reviewers (L.K. and T.C.) using the revised Cochrane Risk of Bias tool for randomized trials (RoB 2) [13]. The RoB 2 tool evaluates potential sources of bias across five key domains: (1) bias arising from the randomization process, (2) bias due to deviations from intended interventions, (3) bias due to missing outcome data, (4) bias in measurement of the outcome, and (5) bias in selection of the reported result.

Each domain was assessed through a series of signaling questions, with reviewers providing responses of “Yes,” “Probably yes,” “Probably no,” “No,” or “No information.” Based on these responses, the RoB 2 tool generated an overall risk of bias judgment for each study, categorized as “Low risk,” “High risk,” or “Some concerns.” All assessments were conducted in accordance with the Cochrane Handbook for Systematic Reviews of Interventions (Higgins et al., 2023) [14].

Disagreements between reviewers were resolved through discussion, and when consensus could not be reached, a third reviewer was consulted.

### Data analysis

All statistical analyses were conducted using Review Manager (RevMan) software, version 5.4. Meta-analyses were performed when two or more studies provided sufficiently homogeneous data for the same outcome measure. For continuous outcomes, mean differences (MD) with 95% confidence intervals (CI) were calculated, depending on whether studies used the same or different measurement scales. For dichotomous outcomes, risk ratios (RR) with 95% CI were computed. Whenever sex-specific data were available, analyses were conducted separately for male and female participants.

A random-effects model using the DerSimonian-Laird method was applied to all meta-analyses to account for anticipated clinical and methodological heterogeneity between studies [15]. The decision to use a random-effects model was made a priori, considering the expected variability in study populations, interventions, and outcome assessment methods.

Statistical heterogeneity was evaluated using the I² statistic and interpreted according to Cochrane Handbook guidelines: 0–40% might not be important; 30–60% may represent moderate heterogeneity; 50–90% may represent substantial heterogeneity; and 75–100% indicates considerable heterogeneity (Deeks et al., 2023) [16]. The Chi² test was also examined, with *P* < 0.10 indicating statistically significant heterogeneity. When substantial heterogeneity (I² ≥ 50%) was detected, potential sources were explored by assessing clinical, methodological, and statistical factors.

For studies that could not be included in meta-analyses due to insufficient data, methodological heterogeneity, or inappropriate study design, a narrative synthesis was conducted following the Synthesis Without Meta-analysis (SWiM) reporting guideline [17].

Results are presented in forest plots with corresponding summary tables. All statistical tests were two-sided, with *P* < 0.05 considered statistically significant.

### Certainty of evidence assessment

Certainty of Evidence Assessment was evaluated using the GRADEpro system (http://www.gradepro.org). Overall certainty of evidence was classified as “High,” “Moderate,” “Low,” or “Very low.” Since this review included RCTs, the initial rating was “High,” which could be downgraded by −1 or −2 points based on the presence of risk of bias, inconsistency, indirect evidence, imprecision, or publication bias.

## Results

### Study selection process

The systematic search across all databases identified 1,202 potentially relevant studies. After removing 113 duplicates using Rayyan 1,089 unique records remained for title and abstract screening. Following independent review by two reviewers, 989 studies were excluded for not meeting the predefined inclusion criteria, leaving 100 studies for full-text assessment.

During the full-text eligibility evaluation, 87 studies were excluded for the following reasons: inappropriate study design (*n* = 45), study protocols without results (*n* = 18), observational studies (*n* = 15), interventions not meeting inclusion criteria (*n* = 6), and insufficient outcome data (*n* = 3).

**Fig. 1:**
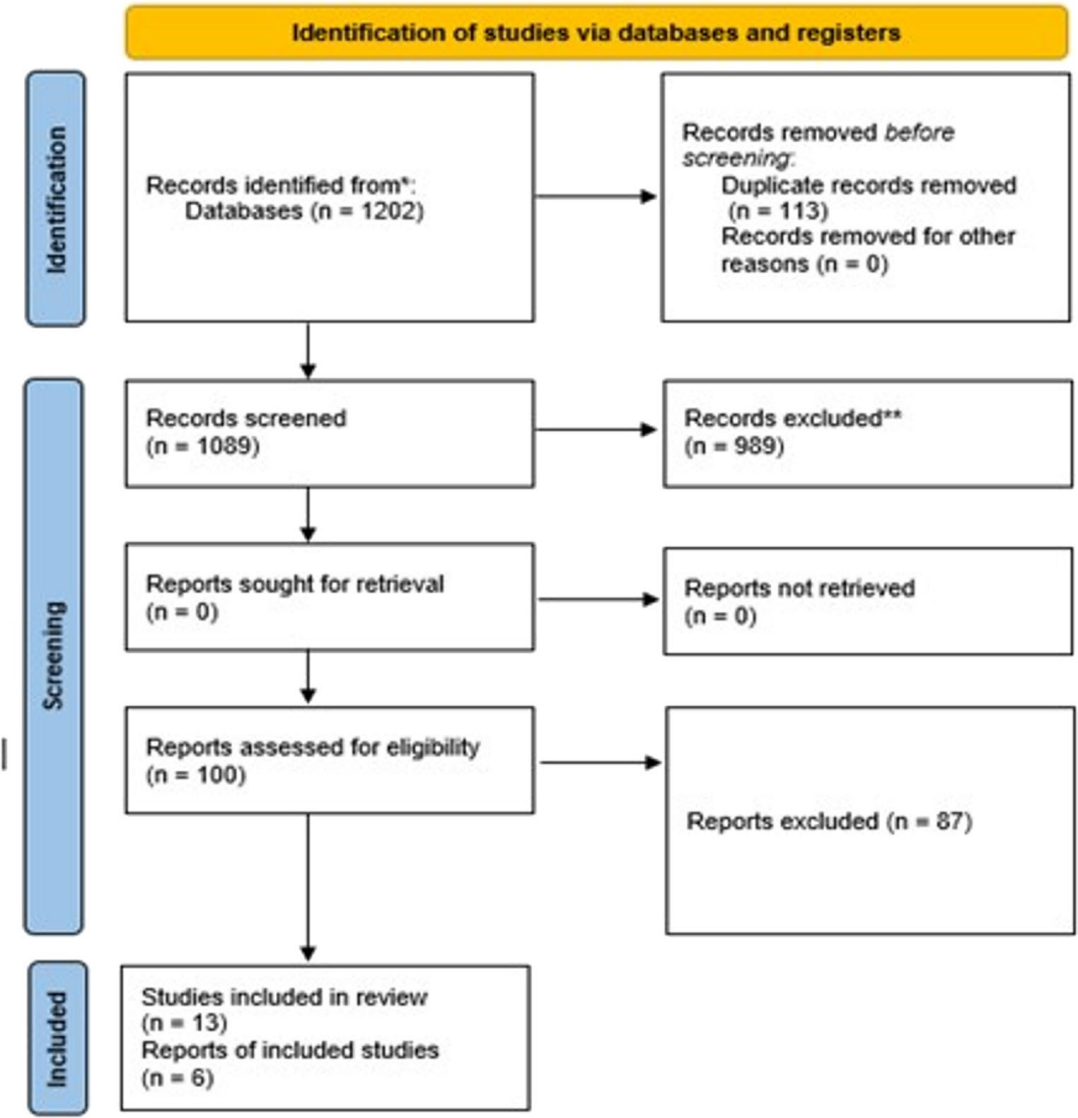
Study selection flowchart

A total of 13 randomized controlled trials met the inclusion criteria and were included in the narrative synthesis [18–30]. Of these, six studies were included in the quantitative synthesis (meta-analysis). The remaining seven studies were excluded from the meta-analysis due to clinical heterogeneity in patient populations (*n* = 3), methodological differences in outcome measurement (*n* = 2), and insufficient statistical data for pooling (*n* = 2). The complete study selection process is illustrated in the PRISMA flow diagram (Fig. 1). Inter-reviewer agreement for study selection was substantial, with Cohen’s kappa coefficients of 0.82 (95% CI: 0.75–0.89) for title/abstract screening and 0.91 (95% CI: 0.84–0.98) for full-text assessment. Disagreements were resolved through discussion, with consultation of a third reviewer when necessary.

### Characteristics of included studies

The 13 included randomized controlled trials were published between 1990 and 2014, with study periods ranging from 1988 to 2013. The trials enrolled a total of 5,941 adult participants (≥ 18 years), including 2,651 men (44.6%) and 3,290 women (55.4%). All participants had a diagnosis of unipolar major depressive disorder based on standardized diagnostic criteria: 11 studies used DSM-IV criteria, one study used DSM-III-R criteria [30], and one study used ICD-10 criteria.

All included studies excluded participants with psychiatric comorbidities such as schizophrenia, bipolar disorder, intellectual disability, personality disorders, obsessive-compulsive disorder, eating disorders (anorexia or bulimia), panic disorder, anxiety disorders, active suicide risk, or substance abuse/dependence within the previous six months (with the exception of nicotine and caffeine).

#### Study settings and duration

The included studies were conducted across multiple countries, with eight studies from the United States, three from Europe (multi-center), and two international multi-center trials. Ten studies were conducted in outpatient settings, two in mixed inpatient/outpatient settings, and one in specialized psychiatric clinics. Treatment duration ranged from 6 to 52 weeks (median: 8 weeks), with follow-up periods extending from 8 to 104 weeks in studies that included post-treatment assessments.

#### Participant characteristics

The mean age of participants across studies ranged from 39.2 to 45.8 years. Baseline depression severity, measured primarily using the Hamilton Depression Rating Scale (HAM-D) or Montgomery-Åsberg Depression Rating Scale (MADRS), indicated moderate to severe depression in most participants (mean baseline scores: HAM-D 18.5–26.2, MADRS 22.1–31.4).

#### Interventions and comparisons

Of the 5,941 total participants, 2,542 (42.8%) received SSRI treatment, while 3,399 (57.2%) were allocated to comparison groups including placebo (*n* = 1,847), active comparators (*n* = 1,234), or other treatment modalities (*n* = 318). The SSRI interventions comprised:

- **Fluoxetine** (20–80 mg/day): evaluated in three studies with 486 participants [19, 24, 26].
- **Paroxetine** (20–50 mg/day): evaluated in three studies with 521 participants [23, 24, 28].
- **Escitalopram** (10–20 mg/day): evaluated in four studies with 732 participants [18, 27, 29].
- **Sertraline** (50–200 mg/day): evaluated in three studies with 567 participants [20, 21, 30].
- **Vilazodone** (20–40 mg/day): evaluated in two studies with 236 participants [22, 25].

#### Outcome measures

Primary efficacy outcomes were assessed using validated depression rating scales, most commonly the 17-item Hamilton Depression Rating Scale (HAM-D; 9 studies) and the Montgomery-Åsberg Depression Rating Scale (MADRS; 4 studies). Secondary outcomes included response rates (typically defined as a ≥ 50% reduction from baseline), remission rates (HAM-D ≤ 7 or MADRS ≤ 10), and measures of functional improvement. Safety outcomes encompassed treatment-emergent adverse events, discontinuation rates, and serious adverse events.

All studies employed appropriate randomization methods, with 11 studies using double-blind designs and two using single-blind methodology. Detailed individual study characteristics are presented in Table 1.

**Table 1:** Characteristics of the included studies

Author, year (country)	Study design	Inclusion criteria	Age: Mean ± SD	Total *N* (*n* per group)	Type of intervention		Control	Treatment session details (duration, quantities)
					Phases (initial and treatment) and duration	Doses (minimum and maximum) and medications/active ingredient		
Clayton et al., 2007 [18] (USA)	RCT	Adults over 18 years old; Diagnosis of MDD according to DSM-IV; Diagnosis by MINI; total score ≥ 22 on MADRS; Score ≥ 4 on CGI-S.	**D**: 41.1 **±** 11.6 **E**: 43.3 **±** 13.0 **Placebo**: 42.5 **±** 12.3	**684** **D**: 273 **E**: 274 **Placebo**: 137 **Women**: **D**: 173 **E**: 186 **Placebo**: 87	**Initial phase (acute)**: 8-week study with fixed dose; **Treatment phase (extension)**: 24-week study with flexible dose.	**Fixed dose:** **D**: 60 mg/day, **E**: 10 mg/day; **Flexible dose:** **D**: 60, 90, or 120 mg/day; **E**: 10–20 mg/day.	**Placebo** (6 capsules to be taken once daily)	**Initial phase (double-blind)**: Lasted 8 weeks with no dose adjustments. **Treatment phase (extension)**: Patients who completed the initial phase participated in the extension phase (open label) lasting 24 weeks, with the possibility of dose adjustments.
Coleman et al., 2001 [19] (USA)	RCT	Adults over 18 years old; Diagnosis of MDD according to DSM-IV; HAM-D item 21 with a minimum score of 20; Normal sexual functioning; sexual activity on 21 occasions every 2 weeks.	**B**: 36.6 **F**: 37.1 **Placebo**: 36.7	**456** **B**: 150; **F**: 154; **Placebo**: 152 **Men**: **B**: 55 (37%) **F**: 53 (34%) **Placebo**: 60 (39%) **Women**: **B**: 95 (63%) **F**: 101 (66%) **Placebo**: 92 (61%)	**Initial phase**: 1 week; **Treatment phase**: 8 weeks.	**Treatment phase:** **B**: 150–400 mg/day or Fluoxetine placebo; **or** **F**: 20–60 mg/day or Bupropion placebo; **or** Fluoxetine placebo/Bupropion SR placebo. **Initial and minimum dose**: **B**: 150 mg/day; **F**: 20 mg/day; **Maximum dose**: **B**: 400 mg/day; **F**: 60 mg/day.	Placebo	**Initial phase**: discontinuation of other medications and identification of patients who no longer met the inclusion criteria; **Treatment phase**: dose adjustments of medications.
Coleman et al., 2001 [20] (USA)	RCT	Adults over 18 years old; Diagnosis of MDD according to DSM-IV; HAM-D item 31 with a minimum score of 18; Depressive episode lasting no more than 2 months; Normal sexual function.	**B**: 38.1 **S**: 38.3 **Placebo**: 38.5	**364** **B**: 122; **S**: 118; **Placebo**: 124 **Men**: **B**: 54 (44%); **S**: 54 (46%); **Placebo**: 51(41%) **Women**: **B**: 68 (56%); **S**: 64 (54%); **Placebo**: 73(59%)	**Initial phase** (screening): 1 week. **Treatment phase**: 8 weeks.	**Treatment phase:** **B**: tablet 150–400 mg/day; or **S**: capsule 50–200 mg/day; or Placebo Sertraline capsule or Placebo Bupropion SR tablet; **Initial and minimum dose**: **B**: 150 mg/day; **S**: 50 mg/day; **Maximum dose**: **B**: 400 mg/day; **S**: 200 mg/day.	Placebo Sertraline capsule; Placebo Bupropion SR tablet. Placebo	**Screening phase**: discontinuation of other medications and identification of patients who no longer met the inclusion criteria; physical examination, vital signs, psychiatric evaluations, assessment of sexual functioning; **Treatment phase**: dose adjustments of medications.
Croft et al., 1999 [21] (USA)	RCT	Adults over 18 years old; Diagnosis of MDD according to DSM-IV; HAM-D item 31 with a minimum score of 18; Major depressive episode lasting 8 weeks to 24 months; Normal sexual function.	**B**: 35.9 **S**: 36.0 **Placebo**: 37.4	**360** **B**: 120 **S**: 119 **Placebo**: 121 **Men**: **B**: 59 (49%) **S**: 60 (50%) **Placebo** **Women**: **B**: 61 (51%) **S**: 59 (50%) **Placebo**: 61(50%)	**Initial phase (screening**): 1 week; **Treatment phase**: 8 weeks.	**Treatment phase**: **B**: 150–400 mg/day or Sertraline placebo; **or** **S**: 50–200 mg/day or Bupropion placebo; **or** Placebo Sertraline or Placebo Bupropion. **Initial and minimum dose**: B: 150 mg/day; S: 50 mg/day; **Maximum dose**: B: 400 mg/day; S: 200 mg/day.	Placebo	**Screening phase**: discontinuation of other medications and identification of patients who no longer met the inclusion criteria; physical examination, vital signs, psychiatric evaluations, assessment of sexual functioning; **Treatment phase**: dose adjustments of medications.
Croft et al., 2014 [22] (USA)	RCT	Adults aged 18 to 70 years; Diagnosis of MDD according to DSM-IV; Major depressive episode lasting ≥ 8 weeks and ≤ 24 months; Normal sexual function; Total MADRS score ≥ 26; Normal clinical results of physical exam, laboratory tests, and ECG; BMI between 18 and 40 kg/m²; Negative β-hCG pregnancy test and use of reliable contraception.	**V**: 39.3 (12.8) **Placebo**: 41.1 (13.2)	**518** **V**: 260 **Placebo**: 258 **Women**: **Placebo: 144** 56.1% **V: 133** 51.4%	**Initial phase (screening)**: 4 weeks; **Treatment phase**: 8 weeks; **Treatment phase with gradual tapering**: 1 week.	**Treatment phase**: **V**: 10 mg/day (week 1); 20 mg/day (week 2); 40 mg/day (weeks 3 to 8). **Treatment phase with gradual tapering**: **V**: 20 mg/day (4 days) and 10 mg/day (3 days).	Placebo	**Screening phase**: medication-free period; **Double-blind treatment phase**: fixed medication dose with gradual increase; **Double-blind treatment phase with gradual tapering**: gradual medication reduction
Dimidjian et al., 2006 [23] (USA)	RCT	Adults aged 18 to 60 years; Diagnosis of MDD according to DSM-IV; Score ≥ 20 on BDI or ≥ 14 on HAM-D item 17.	**N**: 39.90 (10.97)	**241** **P**: 100 **Placebo**: 53 **Cognitive Therapy**: 45 **Behavioral Activation**: 43 **Women**: 159 (66%)	**Triple-blind treatment phase**: 4–8 weeks; **Blind treatment phase**: weeks 8 to 12.	**Triple-blind treatment phase**: **P**: 10–40 mg/day or placebo; **Blind treatment phase (without placebo)**: **P**: 10–50 mg/day.	Placebo	**Triple-blind treatment phase**: gradual medication titration; participants, pharmacotherapists, and evaluators were blinded; **Blind treatment phase**: only evaluators were blinded, and medication dose was flexible.
Fava et al., 1998 [24] (USA)	RCT	Adults over 18 years old; Diagnosis of MDD according to DSM-IV, without history of mania or hypomania; Minimum RDS score of 8; Highest score on Covi Anxiety Scale; Score ≥ 18 on HAM-D item 17.	**N**: 41.3 (12.6)	**128** **P**: 55 **F**: 54 **Placebo**: 19 **Men**:> 63 **Women**: 65	**Initial phase**: 1 week; **Double-blind treatment phase**: 12 weeks.	**Double-blind treatment phase**: **P**: 10–50 mg/day; **F**: 20–80 mg/day.	Placebo	**Initial phase**: medication-free period; **Double-blind treatment phase**: doses could be increased throughout the study, but only once per week.
Khan et al., 2011 [25] (USA)	RCT	Adults aged 18 to 70 years; Diagnosis of MDD according to DSM-IV; Major depressive episode lasting ≥ 4 weeks and < 24 months; Score ≥ 22 on HAM-D item 17.	**V**: 41.1 (12.2) **P**: 42.4 (12.5)	**481** **V**: 235 **P**: 233 **Men (%)**: **V**: 96 (40.9) **P**: 109 (46.8) **Women (%)**: **V**: 139 (59.1) **P**: 124 (53.2)	**Initial phase (screening)**: 4 to 12 weeks; **Double-blind treatment phase**: 8 weeks.	**V**: 10–40 mg/day;	Placebo	**Initial phase (screening)**: washout period from any antidepressant or psychotropic medication (4 weeks for MAOIs or F; 12 weeks for neuroleptics; 2 weeks for other medications); **Double-blind treatment phase**: gradual medication titration with fixed dose.
Michelson et al., 2001 [26] (USA)	RCT	Adults aged 18 to 70 years; Diagnosis of MDD according to DSM-IV; Score ≥ 18 on HAM-D item 17; CGI-S score ≥ 4.	**F 20mg**: 41.7 (11.4) **F 90mg**: 40.9 (11.5) **Placebo**: 42.0 (11.2)	**501** **Men: 159** **Women: 343** **F 20mg**: 189 (H: 55; Women: 134) **F 90mg**: 190 (H: 60; Women: 130) **Placebo**: 122 (H: 44; Women: 78)	**Initial phase (observation)**: 1 week; **Acute open-label treatment phase**: 13 weeks; **Continuation phase**: 25 weeks.	**Initial phase (observation):** **F**: 20 mg/day; **Continuation phase:** **F**: 20 mg/day; **F**: 90 mg/day or placebo)	Placebo	**Initial phase (observation)**: acute treatment phase without blinding; **Continuation phase: double-**blind treatment phase with fixed medication dose.
Nierenberg et al., 2007 [27] (USA)	RCT	Adults over 18 years; Diagnosis of MDD according to DSM-IV; Diagnosis confirmed by MINI; MADRS score ≥ 22; CGI-S score ≥ 4; Normal physical exam, laboratory tests, and ECG results; Negative β-hCG pregnancy test.	**D**: 41.1 (11.6) **E**: 43.4 (13.0) **Placebo**: 42.5 (12.3)	**684** **D**: 273; **E**: 274; **Placebo**: 137 **Women: (%): D**: 173 (63.4); **E**: 186 (67.9); **Placebo**: 87 (63.5) **Men (%)**: 238; **D**: 100; (36.6) **E**: 88 (32,1) **Placebo**: 50 (36.5)	**Acute treatment phase**: 8 weeks; **Continuation phase**: 6 months.	**Acute treatment phase**: **D**: 30 mg, **E**: 10 mg ou **Placebo** (6 capsules)	Placebo	**Acute treatment phase:** Initial and fixed doses; **Continuation phase:** Double-blind treatment.
Perahia et al., 2006 [28] (USA)	RCT	Adults over 18 years; Sites in Bulgaria, Croatia, Hungary, Poland, Romania, Russia, and Slovakia; Diagnosis of MDD according to DSM-IV; Diagnosis confirmed by MINI; CGI-S score ≥ 4; Score ≥ 15 on HAM-D item 17.	**Placebo**: 44.7 (10.1) **D 80mg**: 46.5 (12.7) **D120mg**: 44.0 (10.8) **P**: 45.8 (10.6)	**392** **Placebo**: 99 **D 80mg**: 93 **D 120mg**: 103 **P 20mg**: 97 **N women**: 273 **Placebo**: 65 **D 80mg**: 62 **D 120mg**: 77 **P**: 69 **N man**: 119 **Placebo**: 34 **D** 80 mg: 31 **D** 120 mg: 26 **P**: 28	**Acute treatment phase**: 8 weeks; **Continuation phase**: 6 months.	**Acute treatment phase:** **D**: 80 mg/day (40 mg twice daily); **D**: 120 mg/day (60 mg twice daily); **P**: 20 mg/day or placebo.	Placebo	**Acute treatment phase**: double-blind treatment phase with gradual dose escalation; **Continuation phase**: patients with a ≥ 30% reduction in baseline HAM-D total score.
Pigott et al., 2007 [29] (Canada)	RCT	Adults over 18 years; Diagnosis of MDD according to DSM-IV; Diagnosis confirmed by MINI; MADRS score ≥ 22; CGI-S score ≥ 4.	**D**: 41.1 (11.6) **E**: 43.3 (13.0) **Placebo**: 42.5 (12.3)	**684** **D**: 273 **E**: 274 **Placebo**: 137 **N women: 446** **D**: 173 **E**: 186 **Placebo**: 87 **N men**: 238 **D**: 100 **E**: 88 **Placebo**: 50	**Acute treatment phase**: 8 weeks; **Extension phase**: 24 weeks.	**Acute treatment phase:** **D**: 60 mg/day; **E**: 10 mg/day **or** placebo **Extension phase:** **D**: 60–120 mg/day; **E**: 10–20 mg/day or placebo.	Placebo	**Acute treatment phase**: double-blind treatment phase with initial medications and fixed doses; **Extension phase**: double-blind treatment phase with dose adjustments of medications.
Reimherr et al., 1990 [30] (USA)	RCT	Adults aged 18 to 65 years; Diagnosis of MDD according to DSM-III; Score of 18 on HAM-D item 18; Highest score on RDS.	**S**: Men: 37.9; Women: 40.0; **A**: Men: 39.6; Women: 36.5; **Placebo**: Men: 40.0; Women: 40.2	**448** **Women**: 241 **Men**: 207 **S**: 149 (Men: 70; Women: 79) **A**: 149 (Men:65; Women: 84) **Placebo**: 150 (Men:72; Women: 78)	**Single-blind treatment phase**: 7 to 14 days; **Double-blind treatment phase**: 8 weeks.	**Double-blind treatment phase:** **S**: 50, 100, or 200 mg/day; **A**: 50, 100, or 150 mg/day.	Placebo	**Single-blind treatment phase**: washout of other medications (2 weeks for MAOIs and 1 week for other psychoactive drugs); **Double-blind treatment phase**: medication dose adjustments with a 1-week interval between dose changes.

A: Amitriptyline; B: Bupropion; CGI-S: Clinical Global Impressions Scale– Severy of Illness; CGI-I: Clinical Global Impressions Scale – Improvement of Illness; D: Duloxetine; SD: Standard deviation; DSM: Diagnostic and Statistical Manual of Mental Disorders; RCT: Randomized Clinical Trial; E: Escitalopram; USA: United States; F: Fluoxetine; HAM-D: Hamilton Depression Scale; MAOIs: Monoamine Oxidase Inhibitors; MADRS: Montgomery-Asberg Depression Rating Scale; Mg: milligrams; MINI: Mini-International Neuropsychiatric Interview; N: Nortriptyline; P: Paroxetine;RDS: Raskin Depression Scale; S: Sertraline; TDM: MDD: Major Depressive Disorder; V: Vilazodona

### Evaluation of sexual symptoms using the CSFQ scale

Four studies [18, 25, 27, 29] reported outcomes based on the Changes in Sexual Functioning Questionnaire (CSFQ). The CSFQ is a 14-item self-reported, multiple-choice questionnaire designed to assess sexual functioning, with total and domain scores derived from Likert-scale responses, in which lower scores indicate greater sexual dysfunction (Table 2).

**Table 2:** Assessment of sexual function

Author (Year)	Assessment Tool Used	Sexual desire disorder	Sexual arousal disorder	Orgasm dysfunction	Sexual Satisfaction (during treatment)	Additional Assessment
Clayton, 2006 [18]	Duloxetine 60 mg/day (*N* = 273), escitalopram 10 mg/day (*N* = 274, F 186), or placebo (*N* = 137, F 87) Changes in Sexual Functioning Questionnaire (CSFQ) Categorical changes in sexual functioning were evaluated by analyzing the proportion of patients in each treatment group who showed categorical changes (no change, improvement, or worsening) in the CSFQ score between baseline and endpoint.	Mean difference (SD) – **Week 4**: **Esc**: M 0.01 (0.21); W − 0.17 (0.15) **Pla**: M 0.22 (0.28); W 0.22 (0.21) **Week 8**: **Esc**: M 0.13 (0.23); W 0.07 (0.15) **Pla**: M 0.21 (0.29); W 0.43 (0.23) **Week 32**: **Esc**: M 0.22 (0.32); W 0.11 (0.21) **Pla**: M 0.68 (0.71); W 0.64 (0.50)	Mean difference (SD) – **Week 4**: **Esc**: M − 0.07 (0.27); W − 0.14 (0.18) **Pla**: M 0.65 (0.33); W 0.54 (0.26) **Week 8**: **Esc**: M 0.27 (0.28); W − 0.04 (0.18) **Pla**: M 0.59 (0.35); W 1.00 (0.27) **Week 32**: **Esc**: M 0.56 (0.37); W 0.62 (0.24) **Pla**: M 1.59 (0.76); W 0.73 (0.54)	Mean difference (SD) – **Week 4**: **Esc**: M −0.17 (0.23); W 0.03 (0.15) **Pla**: M 0.50 (0.28); W 0.27 (0.22) **Week 8**: **Esc**: M 0.26 (0.24); W − 0.08 (0.16) **Pla**: M 0.37 (0.30); W 0.52 (0.24) **Week 32**: **Esc**: M 0.57 (0.34); W 0.15 (0.22) **Pla**: M 0.32 (0.73); W 0.94 (0.53)	Mean difference (SD) – **Pleasure** **Week 4**: **Esc**: M −0.08 (0.09); W 0.7 (0.06) **Pla**: M 0.15 (0.12); W 0.28 (0.10) **Week 8**: *P* < 0.05 **Esc**: M 0.24 (0.11); W 0.03 (0.07) **Pla**: M 0.22 (0.14); W 0.37 (0.11) **Week 32**: **Esc**: M 0.68 (0.14); W 0.19 (0.09) **Pla**: M 0.85 (0.33); W 0.44 (0.23)	Mean difference (SD) – **CSFQ** **Week 4**: **Esc**: M −1.09 (0.79); W 0.28 (0.53) **Pla**: M 1.29 (0.99); W 2.01 (0.79) **Week 8**: **Esc**: M 0.08 (0.85); W 0.36 (0.56) **Pla**: M 1.02 (1.07); W 3.42 (0.85) **Week 32**: **Esc**: M 1.34 (1.24); W 1.63 (0.81) **Pla**: M 3.20 (2.62); W 3.53 (1.90) The mean score at baseline was 39.6 for men and 34.1 for women. A total of 449 out of 563 patients (79.8%) met the CSFQ score criteria for overall sexual dysfunction at baseline.
Coleman, 2001 [19]	DSM-IV criteria for sexual dysfunction disorders and Satisfaction scale with overall sexual functioning was assessed by patients using a 6-point Likert-type scale.	Fluoxetine:146 Placebo: 145 **Week 0**: **Flu**: 23% (*n* = 33.58) **Pla**: 13.7% (*n* = 19.86) **Week 4**: **Flu**: 24% (*n* = 35.04) **Pla**: 9.62% (*n* = 13.94)* **Week 8**: **Flu**: 23.9% (*n* = 34.89) **Pla**: 12.8% (*n* = 18.56)*	Fluoxetine:146 Placebo: 145 **Week 0**: **Flu**: 0% **Pla**: 0% **Week 4**: **Flu**: 11,1% (*n* = 16,20) **Pla**: 6% (*n* = 8,7)* **Week 8**: **Flu**: 12% (*n* = 17,52) **Pla**: 9% (*n* = 13,05)	Fluoxetine:146 Placebo: 145 **Week 0**: **Flu**: 0% **Pla**: 0% **Week 4**: **Flu**:28,63% (*n* = 41,79) **Pla**: 9,98% (*n* = 14,47) **Week 8**: **Flu**:30,69%(*n* = 44,80) **Pla**:10,23%(*n* = 14,83)	Fluoxetine:123 Placebo: 120 **Week 0**: **Flu**: 84% (*n* = 123) **Pla**: 83% (*n* = 120) **Week 4**: **Flu**: 71,91% (*n* = 105) **Pla**: 77,24% (*n* = 112)* **Week 8**: **Flu**: 65,06% (*n* = 95) **Pla**: 74,48% (*n* = 108)*	
Coleman, 1999 [20]	DSM-IV criteria for sexual dysfunction disorders and Satisfaction scale with overall sexual functioning was assessed by patients as either satisfactory or unsatisfactory.	Sertraline:109 Placebo: 117 **Day 0**: **Sert**: 31,26% (*n* = 34,07) **Pla**: 32,85% (*n* = 38,43) **Day 21**: **Sert**: 32,21% (*n* = 35,10) **Pla**: 25,87% (*n* = 30,26) **Day 56**: **Sert**: 30,24% (*n* = 32,96) **Pla**: 18,80% (*n* = 21,99		Sertraline:109 Placebo: 117 **Day 0**: **Sert**: 0% **Pla**: 0% **Day 21**: **Sert**:33,06% (*n* = 36,03) **Pla**: 10,99% (*n* = 12,85) **Day 56**: **Sert**:36,09% (*n* = 39,33) **Pla**: 13,79% (*n* = 16,13)	Sertraline:109 Placebo: 117 **Day 0**: **Sert**: 70,27% (*n* = 76,59) **Pla**: 68,63% (*n* = 80,29) *P* < 0,05 **Day 21**: **Sert**: 65,27% (*n* = 71,14) **Pla**: 76,16% (*n* = 89,10) **Day 56**: **Sert**: 62,47% (*n* = 68,09) **Pla**: 80,75% (*n* = 94,47) *P* < 0,05	
Croft, 1999 [21]	DSM-IV e Dichotomized satisfaction	Sertraline:116 Placebo: 116 **Day 0**: **Sert**: 43,34% (*n* = 50,27) **Pla**: 46,0% (*n* = 53,36) **Day 21**: **Sert**: 34,32% (*n* = 39,81) **Pla**: 33,96% (*n* = 39,39) **Day 56**: **Sert**: 28,33% (*n* = 32,86) **Pla**: 31,36% (*n* = 36,37)	Sertraline:116 Placebo: 116 **Day 0**: **Sert**: 0% **Pla**: 0% **Day 21**: **Sert**: 8,13% (*n* = 9,43) **Pla**: 0,97% (*n* = 1,12) *P* < 0,05 **Day 56**: **Sert**:12,27% (*n* = 14,23) **Pla**: 1,19% (*n* = 1,38) *P* < 0,05	Sertraline:116 Placebo: 116 **Day 0**: **Sert**: 0% **Pla**: 0% **Day 21**: **Sert**: 35,52% (*n* = 41,2) **Pla**: 7,72% (*n* = 8,95) *P* < 0,05 **Day 56**: **Sert**:41,77% (n **=** 48,45) **Pla**: 9,14% (*n* = 10,60) *P* < 0,05	Sertraline:116 Placebo: 116 **Day 0**: **Sert**:59,23% (*n* = 68,70) **Pla**: 60,38% (*n* = 70,04) **Day 21**: **Sert** 61,10% (*n* = 70,87) **Pla**: 67,84% (*n* = 78,69) **Day 56**: **Sert**: 65% (*n* = 75,4) **Pla**: 76,92% (*n* = 89,22) *P* < 0,05	
Croft, 2014 [22]				**Week 8**: **Vilazodona**: 4,8% **Pla**: 0,9%		**Week 8**: Delayed ejaculation **Vilazodona**: 2,4% **Pla**: 0,00%
Dimidjian, 2006 [23]	it is unclear how patients were diagnosed	**Week 16**: Paroxetine vs. placebo: 15% versus 0%, 2 (1, *N* = 153) = 8.81, *p* = 0.003;		**Week 16**: Paroxetine vs. Placebo: 17% versus 0%, 2 (1, *N* = 153) = 10.14, *p* = 0.002		
Fava, 1998 [24]	Patient report only And he only reports “sexual dysfunction” without specifying which characteristic it involved, nor providing an evaluation.					At the end of the study, after 12 weeks of follow-up, sexual dysfunction was reported by patients after use of: Paroxetine: 14 patients (25%) *p* < 0.05 Fluoxetine: 4 patients (7%) Placebo: 0 patients
Khan, 2011 [25]	Changes in Sexual Functioning Questionnaire (CSFQ)	Decreased libido was reported by 4.7% of patients taking vilazodone (6 men, 5 women) and by no patients taking placebo.	Sexual dysfunction was more frequent with vilazodone (*n* = 21) than with placebo (*n* = 1).			**Mean CSFQ baseline scores**: **Men**: Vilazodone: 46.5 Placebo: 46.6 **Women**: Vilazodone: 39.4 Placebo: 40.2 **At week 8**,** mean (SD) change in score showing improvement**: **Men**: Vilazodone: 0.6 (7.5) Placebo: 1.8 (6.4) **Women**: Vilazodone: 1.9 (7.9) Placebo: 2.3 (6.2)
Michelson, 2001 [26]	A self-assessment instrument consisting of 4 questions was administered at the beginning of the study, at the end of acute therapy, and at each visit during continuation therapy. The items assessed included sexual interest/desire, erection (for men) or vaginal lubrication (for women), ability to reach orgasm, and an overall rating of sexual function. Each question was rated on a 5-point scale, ranging from 1 (no impairment) to 5 (severely impaired).).	**Acute Treatment**: **Men** FLU 20 mg (*n* = 159): 46.5% improved (*n* = 74); 37.4% no change (*n* = 59); 16.1% worsened (*n* = 26) **Women** FLU 20 mg (*n* = 342): 59.4% improved (*n* = 203); 28.5% no change (*n* = 97); 12.1% worsened (*n* = 41) **Continuation Treatment**: FLU 20 mg (*n* = 189): 26.4% improved (*n* = 50); 47.3% no change (*n* = 89); 26.4% worsened (*n* = 50) **FLU 90 mg** (*n* = 190): 20.1% improved (*n* = 38); 51.6% no change (*n* = 98); 28.3% worsened (*n* = 54) **Placebo** (*n* = 122): 22.2% improved (*n* = 27); 47.0% no change (*n* = 57); 30.8% worsened (*n* = 38)	**Acute Treatment**: **Men** FLU 20 mg (*n* = 159): 29.0% improved (*n* = 46); 56.1% no change (*n* = 89); 14.8% worsened (*n* = 24) **Women** FLU 20 mg (*n* = 342): 33.2% improved (*n* = 114); 56.9% no change (*n* = 195); 9.8% worsened (*n* = 34) **Continuation Treatment**: FLU 20 mg (*n* = 189): 12.7% improved (*n* = 24); 61.9% no change (*n* = 117); 25.4% worsened (*n* = 48) **FLU 90 mg** (*n* = 190): 16.4% improved (*n* = 31); 64.5% no change (*n* = 123); 19.1% worsened (*n* = 36) **Placebo** (*n* = 122): 8.6% improved (*n* = 10); 67.5% no change (*n* = 82); 23.9% worsened (*n* = 29)	**Acute Treatment** (*p* < 0.05) **Men** FLU 20 mg (*n* = 159): 28.6% improved (*n* = 45); 48.7% no change (*n* = 77); 22.7% worsened (*n* = 36) **Women** FLU 20 mg (*n* = 342): 43.8% improved (*n* = 150); 37.9% no change (*n* = 130); 18.3% worsened (*n* = 63) **Continuation Treatment**: FLU 20 mg (*n* = 189): 24.0% improved (*n* = 45); 51.4% no change (*n* = 97); 24.6% worsened (*n* = 46) **FLU 90 mg** (*n* = 190): 19.1% improved (*n* = 36); 60.7% no change (*n* = 115); 20.2% worsened (*n* = 38) **Placebo** (*n* = 122): 16.2% improved (*n* = 20); 57.3% no change (*n* = 70); 26.5% worsened (*n* = 32)	**Acute Treatment**: **Men** FLU 20 mg (*n* = 159): 40.6% improved (*n* = 65); 41.9% no change (*n* = 67); 17.4% worsened (*n* = 28) **Women** FLU 20 mg (*n* = 342): 51.6% improved (*n* = 176); 35.0% no change (*n* = 120); 13.4% worsened (*n* = 46) **Continuation Treatment**: FLU 20 mg (*n* = 189): 20.7% improved (*n* = 39); 53.6% no change (*n* = 101); 25.7% worsened (*n* = 49) **FLU 90 mg** (*n* = 190): 17.5% improved (*n* = 34); 55.2% no change (*n* = 105); 27.3% worsened (*n* = 52) **Placebo** (*n* = 122): 18.0% improved (*n* = 22); 53.0% no change (*n* = 65); 29.1% worsened (*n* = 36)	**Post-randomization groups**: Fluoxetine 20 mg: 55 women and 134 men Fluoxetine 90 mg: 60 women and 130 men Placebo: 44 women and 78 men Change in the sum of the 3 items rating specific aspects of sexual function was highly correlated with change in the item rating overall sexual function during acute therapy (women: *r* = 0.856, *p* < 0.001; men: *r* = 0.925, *p* < 0.001).).
Nierenberg, 2007 [27]	Changes in Sexual Functioning Questionnaire Clinical Version (CSFQ) was collected at Weeks 4 and 8.	Decreased libido 8-Weeks ESC: 11 (4.0%) PLA: 3 (2.2%)				684 patients were randomized to duloxetine 60 mg QD (*N* = 273) Completed 8-weeks acute treatment ITT, *n* = 188 escitalopram 10 mg QD (*N* = 274), Completed 8-weeks acute treatment ITT, *n* = 208 placebo QD (*N* = 137) Completed 8-weeks acute treatment ITT, *n* = 97
Perahia, 2006 [28]	Arizona Sexual Experiences Scale (ASEX) The acute treatment phase lasted 8 weeks, followed by continuation treatment for 6 months. Patients were randomized in a 1:1:1:1 ratio to receive placebo, duloxetine 80 mg/day (administered as 40 mg twice daily [BID]), duloxetine 120 mg/day (administered as 60 mg BID), or paroxetine 20 mg/day.					**Probability of emergent treatment-related dysfunction** **Acute Treatment**: Paroxetine: 64.1% *P* < 0.05 Placebo: 9.6% **Continuation Treatment**: Paroxetine: 24.7% Placebo: 24.7% **Probability of resolution of baseline dysfunction (%)** **Acute Treatment**: Paroxetine: 33.8% Placebo: 26.4% **Continuation Treatment**: Paroxetine: 54.5% Placebo: 39.3%
Pigott, 2007 [29]	Changes in Sexual Functioning Questionnaire (CSFQ) clinical version	**During the Study**: ESC: 18 patients (6.6%) PLA: 4 patients (2.9%)		During the Study: ESC: 11 (4.0%) *P* < 0.05 PLA: 0 (0.0%)	47.4% of male patients treated with escitalopram reported improvement or no change, while 52.6% reported worsening. 66.0% of female patients treated with escitalopram reported improvement or no change, while 34.0% reported worsening.	
Reimherr, 1990 [30]	Patient report			**Men** Sertraline: 21.4% *P* < 0.05 Placebo: 1.4%		

DSM: Diagnostic and Statistical Manual of Mental Disorders; Esc: Escitalopram; Flu: Fluoxetine; M: Men; Pla: Placebo; Sert: Sertraline; W: Women, * = *P* < 0.05 vs. fluoxetine

The total CSFQ cutoff scores for sexual dysfunction were defined as ≤ 47 for men and ≤ 41 for women. However, due to heterogeneity in the data presentation across studies, a meta-analysis could only be performed for two studies [18, 25].

**Fig. 2:**
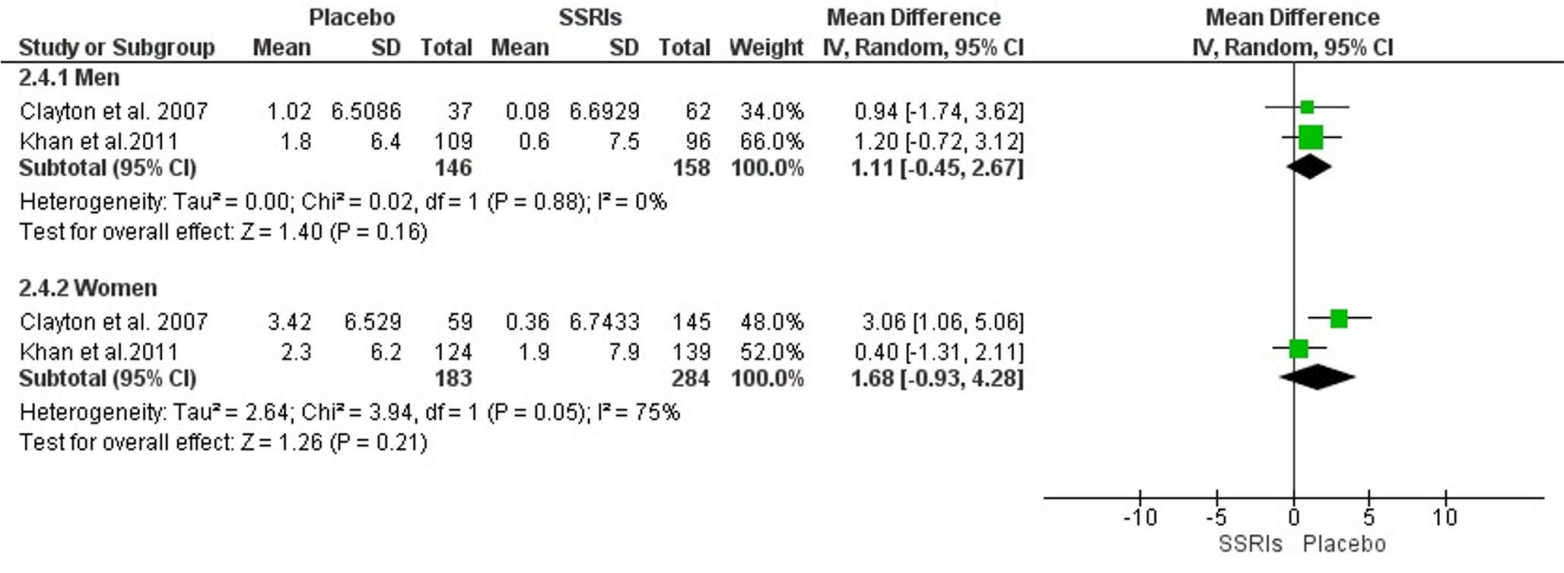
Meta-analysis of the CSFQ scale

At the end of eight weeks of treatment with SSRIs compared to placebo: In men, the mean difference (MD) was − 1.11 (95% Confidence Interval [CI]: −2.67 to 0.45; *p* = 0.16; I² = 0%, 304 participants, two studies, moderate quality of evidence). In women, the MD was − 1.68 (95% CI: −4.28 to 0.93; *p* = 0.21; I² = 75%, 467 participants, two studies, moderate quality of evidence). Accordingly, no statistically significant difference was observed between the groups. These results are illustrated in Fig. 2.

According to GRADE criteria, the certainty of evidence was rated as moderate, downgraded by −1 due to risk of bias (Table 3).

**Table 3:** GRADEpro analysis of SSRIs on CSFQ

GRADEpro analysis of SSRIs versus placebo on CSFQ in adults with depression											
Certainty assessment							Summary of findings				
Participants (studies) Follow-up	Risk of bias	Inconsistency	Indirectness	Imprecision	Publication bias	Overall certainty of evidence	Study event rates (%)		Relative effect (95% CI)	Anticipated absolute effects	
							With placebo	With Score CSFQ 8 weeks		Risk with placebo	Risk difference with Score CSFQ 8 weeks
CSFQ 8-week total score - Men											
304 (2 RCTs)	serious^a^	not serious	not serious	not serious	none	⨁⨁⨁◯ Moderate^a^	146	158	-	146	MD **1.11 lower** (2.67 lower to 0.45 higher)
CSFQ 8-week total score - Women											
467 (2 RCTs)	serious^a^	not serious^b^	not serious	not serious	none	⨁⨁⨁◯ Moderate^a, b^	183	284	-	183	MD **1.68 lower** (4.28 lower to 0.93 higher)

CI: confidence interval; MD: mean difference

*Explanations*

a. High risk of bias in the Clayton 2006 study

b. 75% heterogeneity

### Evaluation of the main sexual side effects

Among the main sexual side effects associated with the use of SSRIs, the most frequently reported were Sexual Desire Disorder, mentioned in 9 studies [18–21, 23, 25–27, 29]; Orgasm Dysfunction, described in 9 studies [18–23, 26, 29, 30]; and Sexual Arousal Disorder, reported in 5 studies [18, 19, 21, 25, 26,].

Sexual satisfaction, as self-reported during the treatment period compared to the pre-treatment period, was assessed in 6 studies [18–21, 26, 29]. Further details related to variations in sexual side effects are presented in Table 2.

### Evaluation of orgasm dysfunction

**Fig. 3:**
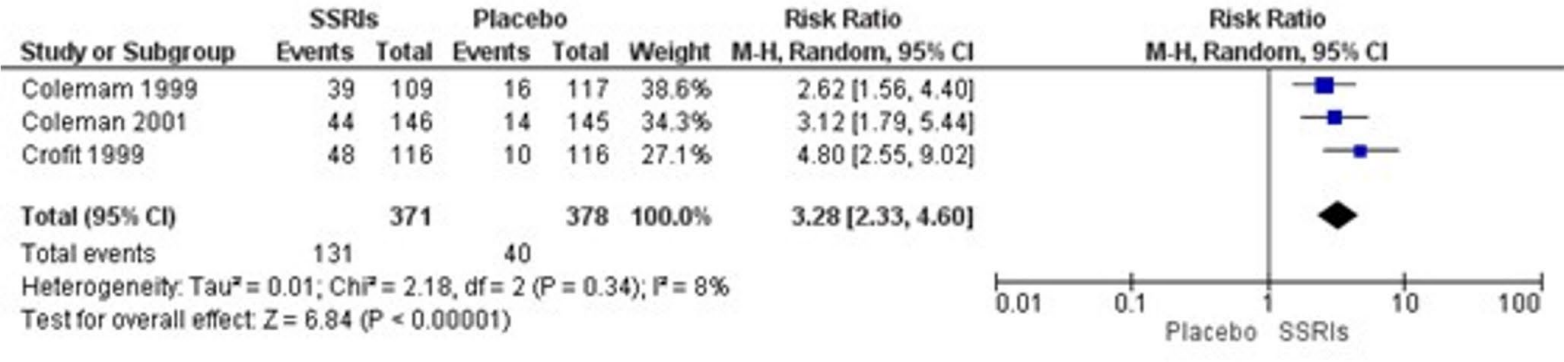
Meta-analysis of orgasm dysfunction disorder

Due to the high variability in data collection across the included studies, a meta-analysis of orgasm dysfunction could only be conducted with 3 studies [19–21]. The analysis revealed a Relative Risk (RR) of 3.28, (95% CI of 2.33 to 4.60, *p* < 0.00001; I² = 8%, 749 participants, three studies, high quality of evidence). A total of 131 events were reported in the SSRI group compared to 40 events in the placebo group, meaning that those who used SSRI had 3.28 times higher risk of having orgasm dysfunction disorder compared to controls (Fig. 3). According to GRADE criteria, the certainty of evidence was rated as high (Table 4).

**Table 4:** GRADEpro analysis SSRIs on sexual desire Disorder, orgasm dysfunction and sexual satisfaction

GRADEpro analysis of SSRIs versus placebo on sexual function in adults with depression											
Certainty assessment							Summary of findings				
Participants (studies) Follow-up	Risk of bias	Inconsistency	Indirectness	Imprecision	Publication bias	Overall certainty of evidence	Study event rates (%)		Relative effect (95% CI)	Anticipated absolute effects	
							With placebo	With SSRIs		Risk with placebo	Risk difference with SSRIsAL
Sexual Desire Disorder											
1199 (4 RCTs)	serious^a^	not serious^b^	not serious	not serious	none	⨁⨁⨁◯ Moderatea, b	79/534 (14.8%)	111/665 (16.7%)	**RR 1.39** (0.93 to 2.08)	79/534 (14.8%)	**58 more per 1000** (from 10 fewer to 160 more)
Orgasm Dysfunction											
788 (3 RCTs)	serious^a^	not serious	not serious	not serious	strong association	⨁⨁⨁⨁ High^a^	40/397 (10.1%)	131/391 (33.5%)	**RR 3.27** (2.28 to 4.70)	40/397 (10.1%)	**229 more per 1000** (from 129 more to 373 more)
Sexual Satisfaction											
788 (3 RCTs)	serious^a^	not serious	not serious	not serious	none	⨁⨁⨁◯ Moderate^a^	238/391 (60.9%)	291/397 (73.3%)	**RR 1.20** (1.09 to 1.33)	238/391 (60.9%)	**122 more per 1000** (from 55 more to 201 more)

CI: confidence interval; RR: risk ratio

*Explanations*

a. Unclear risk of bias in the 1999 Croft study

b. Heterogeneity 51%. Different drugs evaluated, although from the same class. Different criteria used to assess the outcome

### Evaluation of sexual satisfaction

**Fig. 4:**
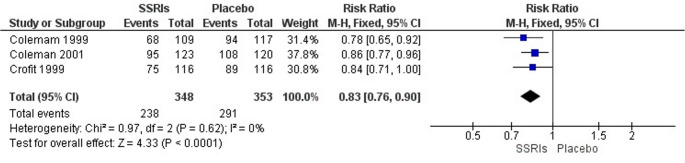
Meta-analysis of sexual satisfaction

To perform the meta-analysis of sexual satisfaction, three studies [19–21] were included. In all included studies, sexual satisfaction was assessed through patient self-report. When SSRIs were compared directly with placebo, the pooled analysis showed a significantly lower likelihood of sexual satisfaction among participants receiving SSRIs (RR = 0.83; 95% CI: 0.76–0.90; *p* = 0.0001; I² = 0%; 701 participants; three studies; moderate certainty of evidence). Overall, sexual satisfaction was reported by 60.8% of participants in the SSRI group (238/348) compared with 73.2% in the placebo group (291/398). These findings indicate that treatment with SSRIs is associated with reduced sexual satisfaction relative to placebo (Fig. 4). According to GRADE criteria, the certainty of evidence was rated as moderate, downgraded by − 1 due to risk of bias (Table 4).

### Evaluation of sexual desire

**Fig. 5:**
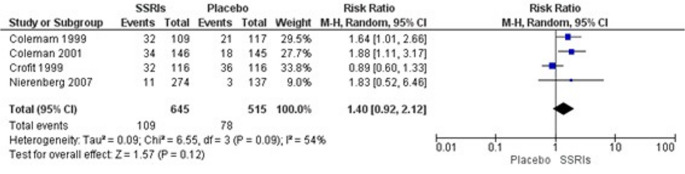
Sexual desire disorder

Meta-analysis incorporating 4 studies [19, 20, 27] showed a trend toward decreased sexual desire in SSRI users (RR = 1.40, 95% CI: 0.92–2.12, *p* = 0.12; I² = 54%, 1,160 participants). However, this difference did not reach statistical significance, though the point estimate suggests a clinically meaningful effect (Fig. 5). According to GRADE criteria, the certainty of evidence was rated as moderate, downgraded by −1 due to risk of bias (Table 4).

### Risk of bias assessment

**Fig. 6:**
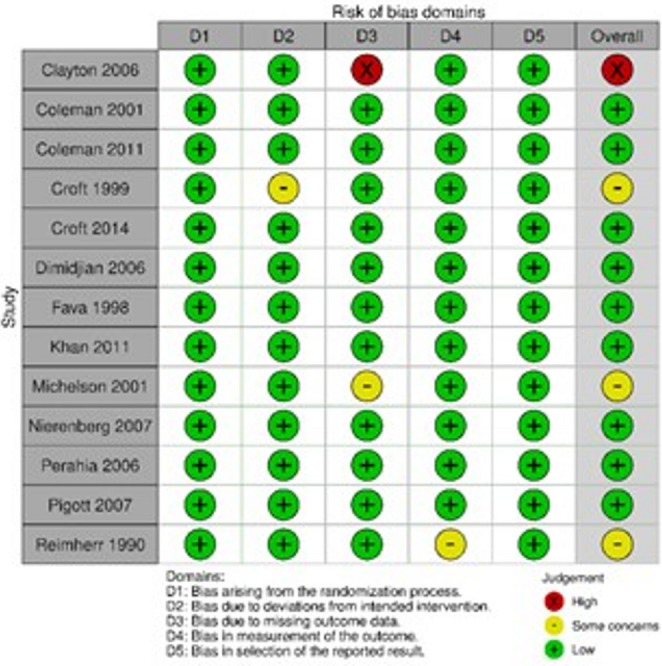
Quality assessment and risk of bias

The risk of bias assessment using the RoB 2 tool revealed that most included studies demonstrated low overall risk of bias, with rigorous randomization processes, adequate allocation concealment, and reliable outcome measurement. However, some studies presented concerns in specific domains. Clayton et al. [18] showed high risk of bias due to missing outcome data, while Croft et al. [21], Michelson et al. [26], and Reimherr et al. [30] raised some concerns regarding deviations from intended interventions or outcome measurement (Fig. 6).

## Discussion

This systematic review and meta-analysis provides robust evidence that SSRI treatment is associated with significant sexual dysfunction in adults with major depressive disorder. Our findings demonstrate statistically significant associations between SSRI use and both orgasmic dysfunction (RR = 3.28) and reduced sexual satisfaction (RR = 1.21). Although sexual desire disorders showed a clinically meaningful trend (RR = 1.40), this did not reach statistical significance, possibly due to heterogeneity in assessment methods and underreporting.

The CSFQ scale analysis, while not reaching statistical significance, consistently showed trends toward poorer sexual functioning in SSRI-treated patients across both genders. The lack of statistical significance may reflect the scale’s limitations in detecting clinically meaningful changes or insufficient power due to the limited number of studies available for analysis.

Our findings align with previous systematic reviews and meta-analyses examining SSRI-associated sexual dysfunction. The observed 3.28 increased risk of orgasmic dysfunction is consistent with clinical reports identifying anorgasmia and delayed orgasm as among the most prevalent and distressing sexual side effects of SSRIs [31]. This substantial effect size reflects the profound impact of serotonergic mechanisms on orgasmic function.

The 21% increased risk of sexual dissatisfaction observed in our analysis corroborates findings from longitudinal studies demonstrating that sexual satisfaction represents one of the domains most significantly affected by long-term SSRI use [32]. This finding is particularly clinically relevant, as sexual satisfaction encompasses the multidimensional impact of sexual dysfunction on overall well-being and relationship quality.

The non-significant trend toward decreased sexual desire (40% increased risk) warrants careful interpretation. While not reaching statistical significance, the effect size suggests clinical relevance and aligns with mechanistic understanding of SSRI effects on libido. The lack of statistical significance may reflect several factors, including underreporting of decreased libido in clinical trials, heterogeneity in assessment methods, and the challenge patients face in attributing reduced sexual desire to medication rather than their underlying depression [33].

The observed sexual dysfunction patterns align with established neurobiological mechanisms of SSRI action. Sexual function involves complex interactions between multiple neurotransmitter systems, with serotonin playing a predominantly inhibitory role. SSRIs increase synaptic serotonin concentrations, leading to enhanced activation of postsynaptic 5-HT receptors, particularly 5-HT2A and 5-HT3 receptors, which are associated with sexual dysfunction [11].

The pronounced effect on orgasmic function likely reflects serotonin’s direct inhibitory effects on spinal ejaculatory reflexes and the neural circuits controlling orgasm. Additionally, increased serotonergic activity can suppress dopaminergic neurotransmission in key brain regions such as the nucleus accumbens and hypothalamus, areas crucial for sexual motivation and arousal [34]. This mechanism may explain the observed trends in sexual desire and arousal, even when not reaching statistical significance in our analysis.

These findings have important implications for clinical practice. Healthcare providers should proactively assess sexual function before initiating SSRI treatment and monitor for sexual side effects throughout treatment. The use of standardized assessment tools, such as the CSFQ or other validated questionnaires, can facilitate systematic evaluation and improve detection rates.

For patients experiencing significant sexual dysfunction, several management strategies warrant consideration. Switching to antidepressants with lower sexual side effect profiles, such as bupropion or mirtazapine, represents a primary approach [35]. Alternatively, adjunctive treatments including phosphodiesterase type 5 inhibitors for erectile dysfunction or orgasmic difficulties may be beneficial [36].

The substantial impact on sexual satisfaction highlights the importance of addressing sexual health as an integral component of depression treatment. Sexual dysfunction can significantly impair quality of life, relationship satisfaction, and treatment adherence, potentially undermining overall therapeutic outcomes.

This systematic review’s strengths include adherence to PRISMA 2020 guidelines, comprehensive database searching, independent reviewer assessment, and use of validated risk of bias and evidence certainty assessment tools. The focus on randomized controlled trials provides high-quality evidence, and the quantitative synthesis allows for precise effect estimates.

However, several limitations should be acknowledged. The heterogeneity in sexual function assessment methods across studies limited our ability to perform comprehensive meta-analyses for all outcomes. Many studies relied on spontaneous adverse event reporting rather than systematic assessment using validated instruments, potentially leading to underestimation of sexual dysfunction prevalence.

The relatively short follow-up periods in most included studies (median 8 weeks) may not capture the full temporal course of sexual side effects or potential adaptation effects. Additionally, most studies excluded patients with comorbidities, limiting generalizability to real-world clinical populations. Moreover, some included trials did not provide sex-disaggregated results, which prevented the conduct of subgroup meta-analyses.

The possibility of publication bias cannot be excluded, as sexual dysfunction may be underreported or inadequately assessed in some clinical trials. Furthermore, the studies included in our analysis were conducted between 1990 and 2014, and contemporary clinical trial practices may differ in their approach to sexual function assessment.

Future research should prioritize the use of standardized, validated sexual function assessment instruments to improve consistency and comparability across studies. Longer follow-up periods are needed to understand the persistence and potential reversibility of sexual side effects, particularly given growing awareness of post-SSRI sexual dysfunction (PSSD).

Comparative effectiveness research examining sexual side effects across different SSRI agents and doses could inform more personalized treatment approaches. Additionally, studies investigating predictors of sexual dysfunction susceptibility could help identify high-risk patients who might benefit from alternative treatment strategies.

Research into management strategies for SSRI-induced sexual dysfunction, including both pharmacological and non-pharmacological interventions, remains a priority. Finally, investigation of the relationship between sexual dysfunction, treatment adherence, and long-term depression outcomes could provide valuable insights for optimizing treatment approaches.

## Conclusion

This systematic review and meta-analysis provide robust evidence that SSRI treatment significantly increases the risk of sexual dysfunction, particularly orgasmic dysfunction and reduced sexual satisfaction. While sexual desire disorders showed a clinically meaningful trend, statistical significance was not achieved, possibly due to methodological limitations in assessment and reporting.

These findings underscore the importance of systematic sexual function assessment and monitoring in patients receiving SSRI treatment. Healthcare providers should engage in open discussions about sexual side effects, consider alternative treatments when appropriate, and implement management strategies to preserve patients’ sexual health and overall quality of life.

The evidence supports the need for continued research using standardized assessment tools and longer follow-up periods to better understand and manage SSRI-induced sexual dysfunction, ultimately improving treatment adherence and patient outcomes in depression care.

## Data Availability

No datasets were generated or analysed during the current study.
